# Association between three-year mortality after transcatheter aortic valve implantation and paravalvular regurgitation graded by videodensitometry in comparison with visual grading

**DOI:** 10.1007/s00392-023-02280-7

**Published:** 2023-08-09

**Authors:** Hesham Elzomor, Timotheus J. Neumann, Linus Boas, Philipp Ruile, Mahmoud Abdelshafy, Ahmed Elkoumy, Pruthvi C. Revaiah, Tsung-Ying Tsai, Klaus Kaier, Osama Soliman, Miroslaw Ferenc, Dirk Westermann, Franz-Josef Neumann, Patrick Serruys, Simon Schoechlin

**Affiliations:** 1 Discipline of Cardiology, Saolta Healthcare Group, Health Service Executive, Galway University Hospital, Galway, Ireland; 2 CORRIB Research Centre for Advanced Imaging and Core Laboratory, Clinical Science Institute, University of Galway, Galway, Ireland; 3 Department of Cardiology and Angiology, University of Freiburg Medical Centre, Südring 15, Bad Krozingen, Germany; 4 Institute of Medical Biometry and Statistics, University of Freiburg Medical Centre, Freiburg, Germany; 5 Faculty of Medicine, University of Freiburg, Freiburg, Germany

**Keywords:** Transcatheter aortic valve implantation, Paravalvular regurgitation, Videodensitometry, Angiography, Mortality

## Abstract

**Background:**

Estimation of regurgitant fraction by videodensitometry (VD-AR) of aortic root angiograms is a new tool for objective grading of paravalvular regurgitation (PVR) after transcatheter aortic valve implantation (TAVI). Stratification with boundaries at 6% and 17% has been proposed to reflect “none/trace”, “mild” and “moderate or higher” PVR.

**Objective:**

We sought to investigate the association of strata of VD-AR with 3-year mortality and to compare VD-AR with visual grading of angiograms.

**Methods:**

We interrogated our database for patients undergoing transfemoral TAVI from 2008 to 2018. Vital status of the patients was obtained from population registers. To test differences in survival and estimate adjusted hazard ratios (HRs) we fitted Cox models.

**Results:**

Our retrospective study included 699 patients with evaluable angiograms at completion of the TAVI procedure. Cumulative 3-year mortality was 35.0% in 261 (37.3%) patients with VD-AR < 6%, 33.9% in 325 (46.5%) patients with VD-AR between 6 and 17% (HR [95% confidence interval] 1.06 [0.80–1.42]; *P* = 0.684) and 47.2% in 113 (16.2%) patients with VD-AR > 17% (HR 1.57 [1.11–2.22]; *P* = 0.011). Visually, PVR was graded as “none/trace” in 470 (67.2%) patients, as “mild” in 219 (31.3%) and as “moderate” in 10 (1.4%). Both mild PVR and moderate PVR on visual grading were significantly associated with mortality (HRs 1.31 [1.12–1.54]; *P* = 0.001 and 1.92 [1.13–3.24]; *P* = 0.015; respectively).

**Conclusions:**

VD-AR > 17%, but not VD-AR 6–17%, was independently associated with mortality. Compared with subjective visual evaluation, VD-AR resulted in a smaller proportion of patients with PVR classified as prognostically relevant.

**Graphical Abstract:**

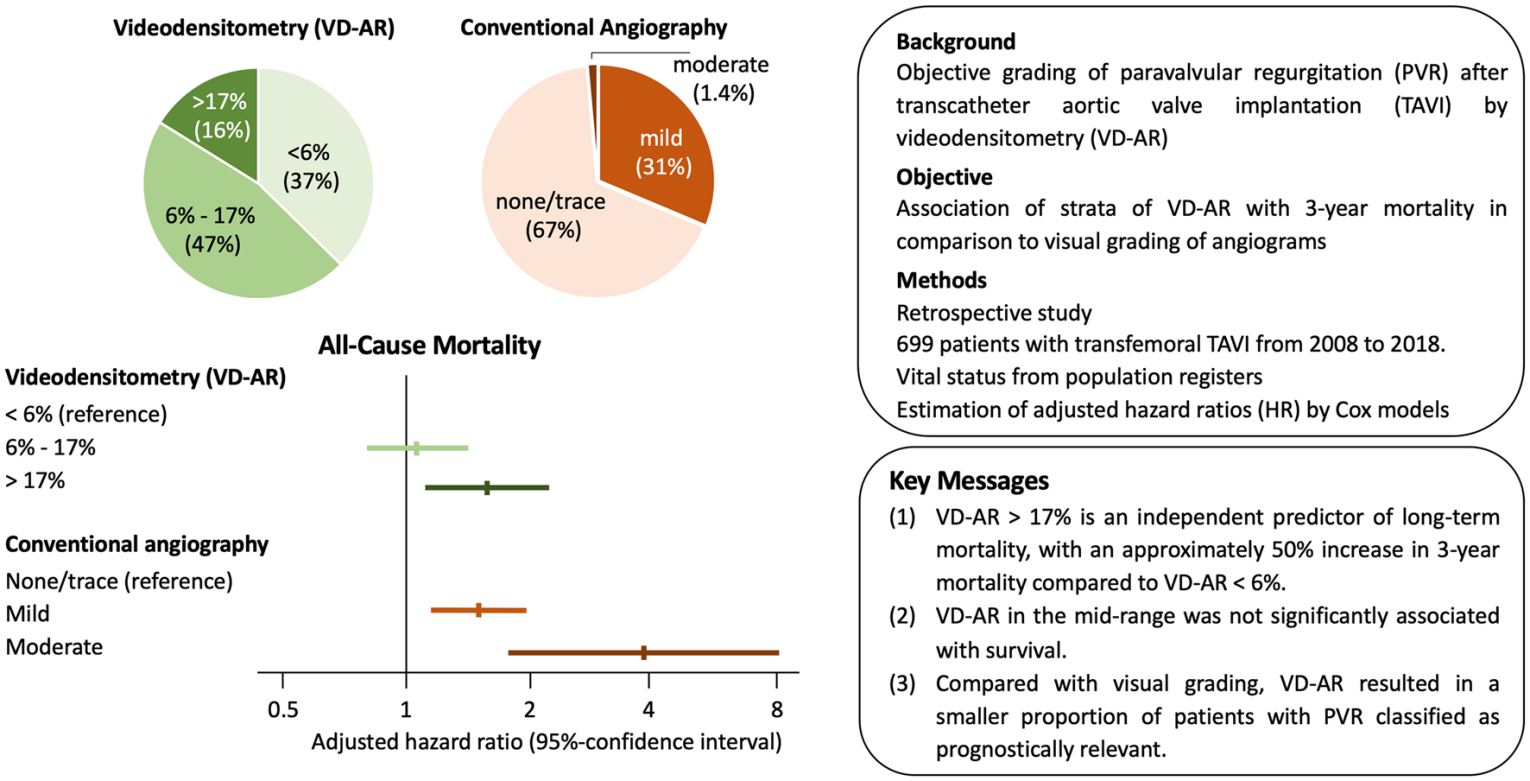

**Supplementary Information:**

The online version contains supplementary material available at 10.1007/s00392-023-02280-7.

## Introduction

After transcatheter aortic valve implantation (TAVI), intraprocedural aortic root angiography frequently reveals some degree of paravalvular regurgitation (PVR). Depending on its severity, PVR affects morbidity and mortality after TAVI. To reduce the PVR immediately after implantation of the transcatheter heart valve (THV), operators may employ additional measures, such as repeated balloon inflation and sometimes use of larger balloons. However, such measures may increase the risk of stroke and even provoke annular rupture.

To resolve the dilemma of whether or not to leave a given PVR, operators must rely on criteria that indicate a prognostically relevant PVR. Based on the grading of aortic valve insufficiency according to Sellers et al. [21], PVR is visually graded on angiography as “none/trace”, “mild”, “moderate” and “severe” [11, 13, 22]. Severe PVR requires immediate repair and there is ample evidence of increased mortality in patients left with moderate PVR after TAVI [5, 6, 9, 12, 13, 18]. Yet, evidence on the prognostic impact of mild PVR is conflicting [6, 9, 12, 13, 18]. This may be due in part to the subjective nature and substantial inter-observer variability of the visual grading of angiograms [10, 14, 20].

To overcome this problem, an objective method for videodensitometric quantification of PVR (VD-AR) has been developed, which measures the ratio of the time-resolved contrast density in the left ventricular outflow tract to that in the aortic root. VD-AR was validated in vitro [2] and in vivo by echocardiography [3] and cardiac magnetic resonance imaging [1]. VD-AR was stratified into < 6%, 6–17% and > 17% corresponding to “none/trace”, “mild” and “moderate” and above according to conventional grading [16, 24, 25]. Analysis of 228 patients of the Brazilian TAVI registry revealed an association of VD-AR > 17% with mortality during a median follow-up of 521 days [23, 24]. Yet, this analysis has not been validated in a large cohort, thus far. Moreover, the prognostic impact of VD-AR in the mid-range remained unclear. To close these gaps in evidence, we interrogated our database of patients undergoing TAVI at our institution.

## Methods

### Patient selection and follow-up

We retrospectively investigated a cohort of patients undergoing TAVI at our institution from June, 2008 to November, 2018. Details of this cohort have been published, previously [19].

In line with contemporary European guidelines [7, 26], patients considered for TAVI had to be assessed by our multidisciplinary team in order to determine eligibility, procedure feasibility, access route, valve type and size. The pre-TAVI assessment included transthoracic and, if necessary, transoesophageal echocardiography as well as computed tomography angiography (CTA). We obtained systolic annular dimensions from planimetric area CTA measurement and effective annulus diameter was calculated, as previously described [8]. TAVI was carried out using general anaesthesia in the majority of cases. The post-interventional antithrombotic treatment usually consisted of dual-antiplatelet therapy with acetyl salicylic acid (100 mg/day) plus clopidogrel (75 mg/day) for 6 months, followed by lifelong acetyl salicylic acid (100 mg/day). In general, those with an indication for oral anticoagulation did not receive concomitant antiplatelet therapy.

As part of our quality assurance programme, we monitor all patients who underwent TAVI by questionnaire or telephone call at 30 days, 6 months and 1 year after the procedure, and then annually for 5 years. To complement the survival data, we interrogated the register of death.

This study was approved by the Institutional Clinical Research and Ethics Committee (registration number 21-1623).

### Conventional grading of PVR

Unless contraindicated by renal failure, we routinely performed aortic root angiography after THV implantation. If needed, this was repeated after further optimisation of THV sealing. The pigtail catheter was placed 2–3 cm above the THV cusps. In general, we injected 36 ml of contrast agent at a speed of 18 ml/s or less if clinically indicated. In the present study, only the final angiograms were evaluated visually and by videodensitometry (see below). By visual assessment, PVR was graded intraprocedurally as follows: “none/trace”, “mild” with reflow of contrast in the outflow tract and middle portion of the LV but clearing with each beat, “moderate” with reflow of contrast in the whole left ventricular cavity with incomplete washout in a single beat and faint opacification of the entire LV over several cardiac cycles and “severe” with opacification of the entire LV with the same intensity as in the aorta and persistence of the contrast after a single beat [22].

Transthoracic echocardiography was scheduled before discharge. In general, two independent echocardiographers not having attended the procedure performed the echocardiography and differences were settled by consensus. In accordance with the valve academic research consortium-two (VARC-2) criteria, PVR was graded as “none/trace”, “mild”, “moderate” and “severe” [11].

### Videodensitometry

Quantitative assessment of PVR by videodensitometry was performed as described previously [1, 2, 20, 24]. In brief, the software (CAAS A-valve version 2.0.2 – research mode, Pie Medical Imaging, Maastricht, The Netherlands) calculated time–density curves in the aortic root and in the left ventricular outflow tract. The ratio of the integral of these 2 curves is the continuous regurgitant fraction VD-AR, expressed as a percentage. Pseudonymised angiograms were analysed by an independent academic core laboratory in Galway, Ireland, with operators blinded to the clinical data.

### Statistical analysis

Discrete variables are presented as counts (percentages) and compared using the *χ*^2^ test. We checked continuous variables for normal distribution using the Kolmogorov–Smirnov test. Depending on the result of this test, continuous variables are reported as mean ± standard deviation or median (25th to 75th percentile) and compared using the Student’s *t*-test or the Mann–Whitney *U* test, respectively.

Cumulative 3-year mortality was analysed and visualised by the Kaplan–Meier method. Patients with incomplete follow-up were censored at the time of the last contact, the rest of the cohort at 1095 days. To test differences in survival and estimate crude and adjusted hazard ratios (HR) with 95% confidence intervals (CI), we fitted univariable and multivariable Cox proportional regression models. Variables in Tables 1 and 2 that differed between survivors and non-survivors with *P* < 0.1 were included in the multivariable Cox models. Some of the variables shown in Table 1 are included in the EuroSCORE and the direction of association with mortality of a single variable of the EuroSCORE may differ from that of the EuroSCORE as a summary variable. Therefore, the individual variables that constitute the EuroSCORE were entered into the multivariable Cox models instead of the EuroSCORE itself. To account for potential temporal changes in mortality after TAVI, we also included the time period when TAVI was performed as an additional variable in the multivariable Cox models. For this purpose, we considered an early period from 2008 to 2013 and a late period from 2014 to 2018. In addition to fitting Cox models, we performed receiver-operator curve analyses to calculate the area under the curve (AUC).

**Table 1:** Baseline characteristics

	All patients (*n* = 699)	Survivors (*n* = 445)	Non-survivors (*n* = 254)	*P* value
Age, years	83 (80, 87)	83 (80, 86.5)	84 (79, 87)	0.417
Female	391 (55.9)	261 (58.7)	130 (51.2)	0.056
Logistic EuroSCORE, %	16.4 (9.5, 27.6)	14.9 (8.7, 14.9)	19 (11.0, 33.3)	< 0.001
Mean aortic gradient, mmHg	42.5 (33, 53)	44 (33.3, 54.8)	40 (27, 51)	0.004
LVEF, %	60 (45, 60)	60 (45, 60)	60 (42, 60)	0.021
Hypertension	624 (89.3)	403 (90.6)	221 (87.0)	0.144
Diabetes mellitus	192 (27.5)	117 (26.3)	75 (29.5)	0.357
Glomerular filtration rate	47.7 (35.7, 60.6)	52.3 (39.3, 67.6)	42.2 (30.4, 58.9)	< 0.001
Coronary artery disease	440 (62.9)	277 (62.2)	163 (64.2)	0.612
Peripheral artery disease	86 (12.3)	49 (11.0)	37 (14.6)	0.169
Cerebrovascular disease	131 (18.7)	75 (16.9)	56 (22.0)	0.091
Pulmonary hypertension	365 (52.2)	229 (51.5)	136 (53.5)	0.596
Previous myocardial infarction	106 (15.2)	63 (14.2)	43 (16.9)	0.326
Previous CABG	66 (9.4)	34 (7.6)	32 (12.6)	0.031
Previous aortic valve surgery	13 (1.9)	7 (1.6)	5 (2.0)	0.458

Values are median (interquartile range) or counts (percentage of column)

*CABG* coronary artery bypass graft, *EuroSCORE* European System for Cardiac Operative Risk Evaluation, *LVEF* left ventricular ejection fraction, *mmHg* millimetre of mercury

**Table 2:** Procedural characteristics

	All patients (*n* = 699)	Survivors (*n* = 445)	Non-survivors (*n* = 254)	*P* value
TAVI during early period (2008–2013)	238 (34.0)	129 (29.0)	109 (42.9)	< 0.001
Dilatation				
Predilatation	213 (30.5)	120 (27.0)	93 (36.6)	0.008
Postdilatation	135 (19.3)	89 (20.0)	46 (18.1)	0.543
Valve type				0.008
Balloon-expandable	532 (76.1)	354 (79.6)	178 (70.1)	
Self-expandable	154 (22.0)	86 (19.3)	68 (26.8)	
Mechanically expandable	13 (1.9)	5 (1.1)	8 (3.1)	

Values are counts (percentage of column)

In the 2-sided test, a *P* value < 0.05 was regarded as significant. All statistical analyses were performed using SPSS version 28.0 (SPSS, Chicago, Illinois, United states).

## Results

### Study population

Out of 2129 consecutive patients undergoing TAVI from June 2008 to November 2018, 699 (32.8%) had analysable angiograms for VD-AR calculation. These patients constitute the study cohort. In 1430 patients, final angiography was either not performed or the angiogram could not be analysed to measure VD-AR because of overlapping spine, overlapping descending aorta, overlapping breathing motion TEE probe, overlapping electrode leads or other dense objects, low position of the pigtail catheter, arrhythmia, insufficient image acquisition (less than 2 cardiac cycles before contrast injection) or inadequate opacification due to low contrast volume.

There were no major differences in baseline characteristics between patients with and without videodensitometry (Supplemental Table). Albeit statistically significant, the differences in mean aortic gradient and left ventricular ejection fraction were minimal, as was the difference in the proportion of patients with diabetes. In patients with VD-AR, balloon-expandable THVs were used more frequently at the expense of fewer self-expanding THVs in patients without VD-AR. Predilatation was also performed more often in patients with VD-AR. The 3-year mortality in patients with VD-AR was 36.3% and was not statistically significantly different from the mortality of patients without VD-AR.

Vital status at 3 years after TAVI could be obtained in all but 12 patients, due to blocked register entry or foreign residency. The baseline characteristics of the study cohort are shown in Table 1. Median age was 83 (quartiles 80, 87) years and 391 (55.9%) were female. Median logistic EuroSCORE was 16.4 (quartiles 9.5, 27.6). As shown in Table 2, balloon-expandable THVs were implanted most frequently, followed by self-expandable THVs. In 69.5% of the patients THVs were implanted without predilatation and postdilatation was performed in only 19.3%.

During 3-year follow-up, 254 (36.3%) patients died. Survivors and non-survivors differed significantly in several baseline characteristics, as shown in Table 1: These were a higher logistic EuroSCORE, a lower mean aortic gradient, a lower left ventricular ejection fraction, a lower glomerular filtration rate and a higher proportion of patients with previous coronary artery bypass grafting in non-survivors compared with survivors. There were also differences in THV type between non-survivors compared with survivors and predilatation had been performed more often in non-survivors than in survivors. Furthermore, the group of non-survivors included a significantly higher proportion of patients whose intervention was performed in the early period from 2008 to 2013 (Table 2).

### Distribution of VD-AR

**Fig. 1:**
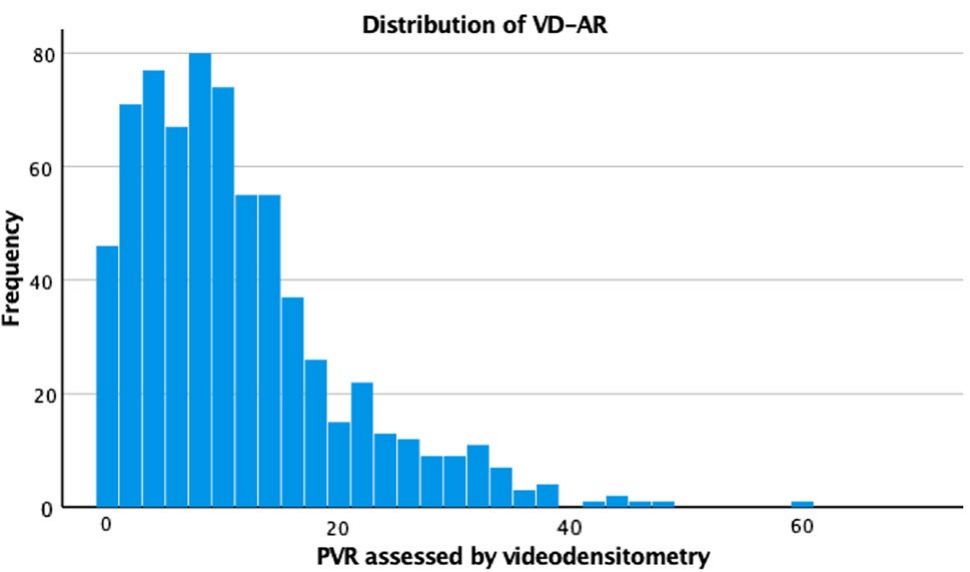
Distribution of paravalvular regurgitation assessed by videodensitometry (VD-AR)

**Fig. 2:**
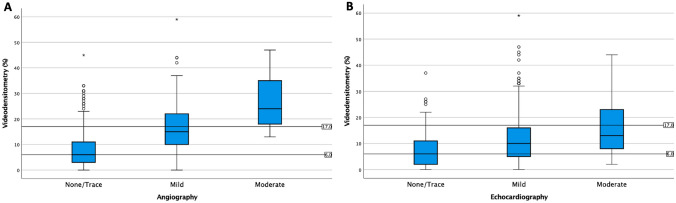
Box and whisker representation of paravalvular regurgitation (PVR) measured by videodensitometry (VD-AR) versus angiographic grades assessed visually (**A**) and echocardiographic assessment before discharge (**B**)

This distribution of VD-AR is shown in Fig. 1 for the whole cohort and according to valve type in Supplemental Fig. 1. VD-AR was < 6% in 261 (37.3%) patients, between 6 and 17% in 325 (46.5) and > 17% in 113 (16.2%) (Graphical Abstract, Table 3). By visual assessment of aortic root angiograms PVR was graded as “none/trace” in 470 (67.2%) patients, as “mild” in 219 (31.3%) and as “moderate” in 10 (1.4%) patients (Graphical Abstract, Table 3). The distribution of VD-AR in these strata is shown in Fig. 2A and Table 3. Among patients with none/trace PVR by visual assessment 207 (44%) had an intermediate VD-AR and 27 (5.7%) a VD-AR in the high range. In those with mild PVR by visual assessment, 79 (36.1%) were in the high range of VD-AR. Conversely, 3 out of 10 angiograms graded as moderate PVR visually were in the mid-range of VD-AR and 25 (9.6%) visually graded as mild were in the low range of VD-AR. The strata of VD-AR were concordant with the visual estimates in 358 (51.2%) patients.

**Table 3:** Grading by VD-AR versus grading by angiography or echocardiography

		Angiography (*n* = 699)			Echocardiography (*n* = 680)		
		None/Trace	Mild	Moderate	None/Trace	Mild	Moderate
VD-AR							
< 6%	261 (37.3)	236 (50.2)	25 (11.4)	0 (0)	117 (53.2)	134 (32.1)	7 (16.3)
6–17%	325 (46.5)	207 (44)	115 (52.5)	3 (30)	93 (42.3)	200 (48)	19 (44.2)
> 17%	113 (16.2)	27 (5.7)	79 (36.1)	7 (70)	10 (4.5)	83 (19.9)	17 (39.5)
In total	699 (100)	470 (100)	219 (100)	10 (100)	220 (100)	417 (100)	43 (100)

Values are counts (percentage of column). Echocardiographic assessment before discharge

*VD-AR* Videodensitometric assessment of aortic regurgitation

Assessment of PVR by transthoracic echocardiography at discharge was available in 680 patients. Out of these, 220 (32.4%) were graded as none/trace, 417 (61.3%) as mild and 43 (6.3%) as moderate PVR (Graphical Abstract, Table 3). Figure 2B shows the distribution of VD-AR in these strata. Grading was concordant between echocardiography and videodensitometry in 334 (49.1%) patients, whereas videodensitometry resulted in a higher PVR category in 186 (27.4%) patients and in a lower category in 160 (23.5%) patients (Table 3).

### Prognostic relevance of PVR grades

**Fig. 3:**
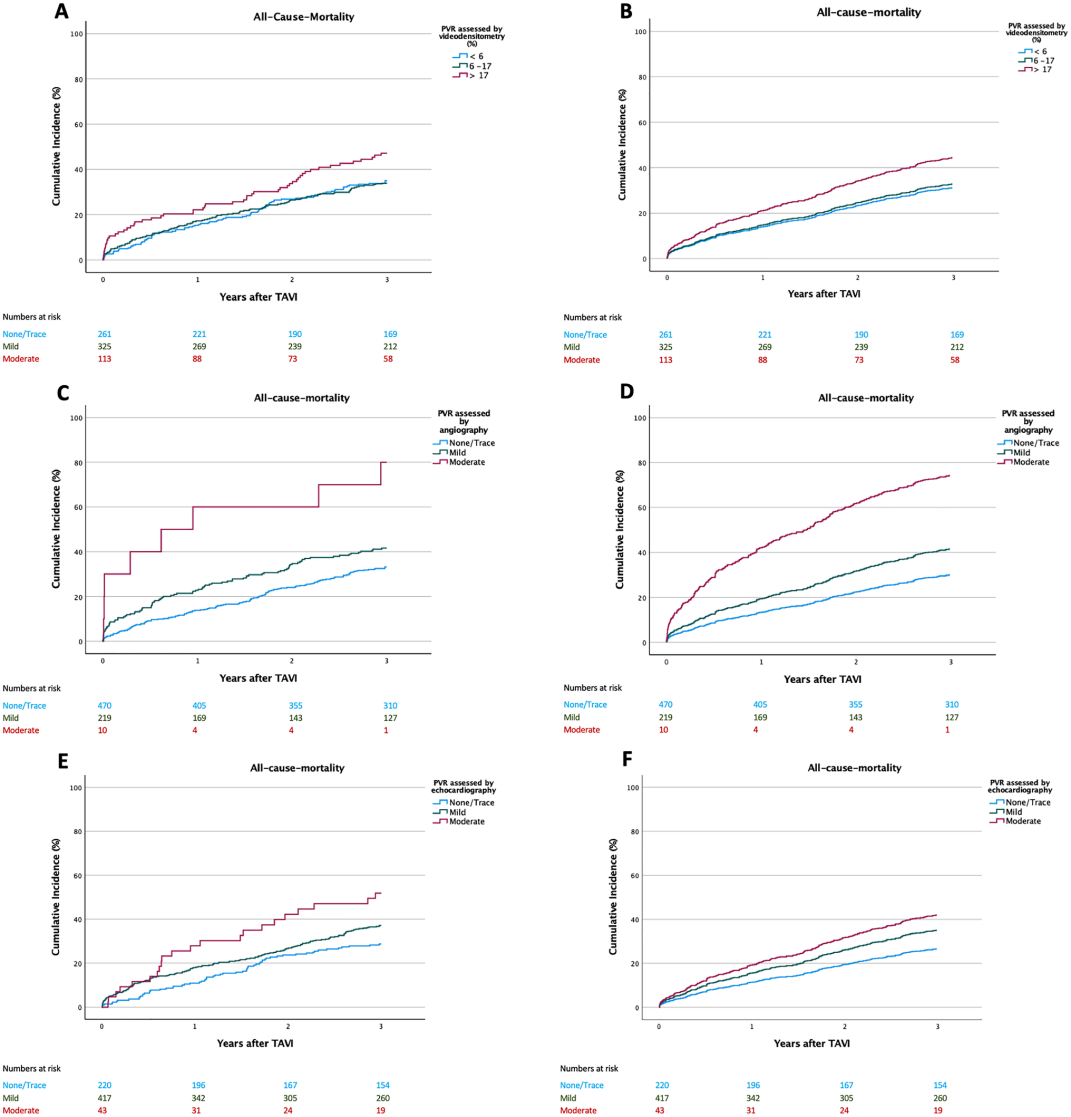
Kaplan–Meier curves and adjusted incidence curves from Cox models for cumulative all-cause mortality according to grades of paravalvular regurgitation (PVR) assessed by videodensitometry (**A, B**), angiographic grades assessed visually (**C, D**) and pre-discharge echocardiographic grades (**E, F**)

Strata of VD-AR were significantly associated with 3-year mortality in both the crude and the adjusted analysis (*P* = 0.026 and *P* < 0.001, respectively). Crude and adjusted survival curves are shown in Fig. 3A and B. During 3-year follow-up, there were 91 deaths in the low stratum of VD-AR, 110 in the intermediate stratum and 59 in high stratum, corresponding to a cumulative 3-year mortalities of 35.0%, 33.9% and 47.2%, respectively. Three-year mortality was significantly increased in patients with VD-AR > 17% as compared to patients with VD-AR < 6% (Graphical Abstract, Table 4). However, neither in the crude nor in the adjusted analysis did we find a significantly increased 3-year mortality in patients with intermediate VD-AR (Table 4). The AUC for VD-AR was 0.533.

**Table 4:** Crude and adjusted hazard ratios for all-cause mortality by grades of PVR assessed through videodensitometry, conventional angiography and echocardiography

Grading	Crude		Adjusted	
	HR (95% CI)	*P* value	HR (95% CI)	*P* value
Videodensitometry				
< 6%	Reference		Reference	
6–17%	0.98 (0.74–1.29)	0.871	1.06 (0.80–1.42)	0.684
> 17%	1.50 (1.06–2.09)	0.021	1.57 (1.11–2.22)	0.011
Angiography				
None/trace	Reference		Reference	
Mild	1.39 (1.07–1.80)	0.012	1.50 (1.15–1.96)	0.003
Moderate	4.14 (2.03–8.44)	< 0.001	3.79 (1.77–8.14)	< 0.001
Echocardiography				
None/trace	Reference		Reference	
Mild	1.37 (1.02–1.84)	0.034	1.40 (1.03–1.90)	0.033
Moderate	2.15 (1.32–3.50)	0.002	1.76 (1.05–2.94)	0.031

*HR* Hazard ratio, *CI* Confidence interval

As shown in Fig. 3C–F and Table 4, 3-year mortality differed significantly with the grade of PVR either assessed by conventional angiography at the end of the procedure or by transthoracic echocardiography before discharge. With both grading techniques, even mild PVR was significantly associated with increased 3-year mortality in the crude and adjusted analysis. An even stronger impact on 3-year mortality was observed in patients with moderate PVR irrespective of whether assessed by on conventional angiography or transthoracic echocardiography (Table 4). The AUCs for conventional angiography at the end of the procedure and for transthoracic echocardiography before discharge were 0.553 and 0.559, respectively.

## Discussion

To the best of our knowledge, our study is the largest to evaluate the association of PVR objectively assessed by videodensitometry with survival after TAVI and the first to specifically address the prognostic relevance of intermediate VD-AR suggestive of mild PVR. Our main findings are as follows: (1) VD-AR > 17% is a significant, independent predictor of long-term mortality, with an approximately 50% increase in 3-year mortality compared to VD-AR < 6%, indicating trivial or no PVR. (2) Compared to VD-AR < 6%, VD-AR in the mid-range of 6–17% was not significantly associated with survival in either crude or adjusted analyses.

Grades of PVR assessed by conventional visual evaluation of aortic root angiograms or by transthoracic echocardiography were also predictive of 3-year mortality. However, grades of PVR assessed by conventional angiography or echocardiography matched the strata of VD-AR in only about half of the cohort. Compared with VD-AR, conventional grading was more likely to result in a lower category of PVR than in a higher category. Comparison of the associations of mortality with the PVR grades obtained from different methods suggests that, with current definitions, the range of PVR covered by the conventional grade “mild” includes PVRs that are prognostically irrelevant as well as those with an impact on survival. Conversely, the conventional grade of “moderate” appears to refer only to those PVRs within the range of prognostically relevant PVRs that carry a very high (echocardiography) or even extreme risk (angiography). Apart from different thresholds, the subjective nature of conventional grading with a potential bias towards lower grades, as well as the notorious inter-observer variability, would have contributed to the discrepancy between VD-AR and conventional grading of PVR [10, 14, 20].

In addition to conventional angiography and echocardiography, haemodynamic assessment, such as aortic regurgitation index (ARI) [22], may be considered in relation to VD-AR. However, in our TAVI cohort neither ARI nor ARI ratio was predictive of mortality [18] and the same was true for the current subset (data not shown).

### Comparison with previous studies

The present study validates previous findings on the prognostic relevance of VD-AR > 17%. [4, 23, 24]. Previously, 228 patients from the Brazilian TAVI Registry (Interaction of de novo) were assessed for PVR by videodensitometry and followed for up to 3 years with a median of 521 days. Of these, 73 (32%) patients had a VD-AR > 17%. The estimated 3-year mortality was 45.5% in patients with VD-AR > 17% compared to 37.7% (log rank *P* = 0.036), corresponding to an adjusted HR of 1.73 (95% CI 1.05–2.86; *P* = 0.032). However, 3-year survival estimates were imprecise due to the small number of patients with follow-up beyond 18 months. With nearly complete 3-year follow-up and three times the number of patients, the current study provides more robust data. It validates the previous findings with similar survival rates and similar HRs for patients with a VD-AR > 17%. As an important new aspect, the present study addresses the role of VD-AR in the mid-range, which is considered to correspond to mild PVR by conventional angiographic or echocardiographic assessment. It refutes an association of intermediate VD-AR with increased mortality and thus strengthens the cut-off of VD > 17% as an identifier of prognostically relevant PVR.

### Limitations

Although VD-AR, conventional angiography and echocardiography reliably identified subgroups at increased risk as shown by the Cox models, the C-statistics demonstrated that the identification of individual patients who will die was poor with each of these measures of PVR. The benefit of VD-AR over the other measures of PVR was not in improving individual prediction, but in more distinctly defining the risk group based on PVR.

The retrospective, observational design is a limitation of this study. Although we fitted Cox models to adjust for baseline and procedural characteristics, we cannot exclude unknown confounders. Moreover, angiograms in this retrospective cohort were not optimised for the assessment of VD-AR. Thus, VD-AR was available in only one-third of the original cohort, whereas a recent study with prospective optimisation of angiograms for videodensitometry reported readability in 95% [15]. Nevertheless, patients with VD-AR readout may be considered largely representative of the entire cohort as we found no major differences in baseline characteristics or survival between those with and without VD-AR measurement.

To obtain complete information on the vital status we had to rely on public records that do not provide information on the cause of death. Therefore, we could not distinguish between cardiac and non-cardiac causes of death. The unknown admixture of non-cardiac deaths confounds the relationship between PVR and mortality. It is, however, reasonable to assume that the prognostic impact of PVR is at least as strong as that observed in this study.

Apart from mortality, VD-AR > 17% may be associated with repeat hospitalisation for heart failure, as suggested by a previous study of 51 patients [24]. However, in the present study, complete data on repeat hospitalisation were not available for 3-year follow-up. Therefore, the current analysis did not address this issue.

Moreover, off-line core lab calculation of VD-AR compared with intraprocedural qualitative operator assessment may have penalised conventional grading. However, there is no alternative to operator judgement when it comes to clinical decision making for additional interventions to improve THV sealing. Alternatively, an online version of videodensitometry is available. A previous study found high agreement between online and the core laboratory calculation of VD-AR [17].

Our study was not designed to compare different THVs. THVs were selected according to the clinical conditions. The observed associations between THV types and mortality, therefore, need to be interpreted cautiously. They cannot be considered specific to each THV, but rather to the subset of patients for whom that THV was chosen.

### Implications for clinical practice

Currently, most TAVIs are performed without general anaesthesia. In this setting, the image quality of transthoracic echocardiography is often poor due to suboptimal patient positioning and transoesophageal echocardiography is too stressful for the patient. Therefore, in TAVI without general anaesthesia, operators must rely on angiography when deciding on the need for additional measures to reduce PVR.

Although both conventional and videodensitometric grading of PVR provide relevant prognostic information, videodensitometry offers several advantages for clinical practice. As established by previous studies, VD-AR is an operator independent, highly reproducible, objective measure of PVR [2, 17, 24] and, therefore, provides the best basis not only for unbiased clinical decision making, but also for further scientific evaluation. More importantly, the present study suggests that VD-AR improves intraprocedural identification of PVR requiring correction. With conventional angiographic assessment, even PVR graded as “mild” would require further intervention, because the whole stratum carries an increased risk of death. With VD-AR, however, some of these “mild” PVRs fall in the intermediate range of VD-AR without prognostic relevance, thus obviating the need for additional intervention, as shown by the current data. Hence, application of the threshold of VD-AR > 17% may avoid potentially harmful extension of the procedure and limit additional measures to reduce PVR to those patients who may benefit. Given the limitations of a retrospective observational study, this concept needs to be confirmed by further research. In this respect, the present study breaks the ground for a randomised trial comparing the outcomes of TAVI guided by videodensitometry for assessment of PVR with those of TAVI guided by conventional angiography.

### Supplementary Information

Below is the link to the electronic supplementary material.

Supplementary file1 (DOCX 5217 KB)

## Data Availability

Data is available on author's request.
