# Supplementary material for: Association between three-year mortality after transcatheter aortic valve implantation and paravalvular regurgitation graded by videodensitometry in comparison with visual grading
Source: Clin Res Cardiol. 2023 Aug 9;113(1):116–25. doi: 10.1007/s00392-023-02280-7 (PMC10808559; doi:10.1007/s00392-023-02280-7)
Supplement: Supplementary file 1 — Supplementary file1 (DOCX 5217 KB) [file 392_2023_2280_MOESM1_ESM.docx]

| **Supplemental table.** |  |  |  |  |
| --- | --- | --- | --- | --- |
| *Baseline and procedural characteristics of patients with and without VD-AR assessment* | | | |  |
|  | **All Patients (n=2129)** | **With (n=699)** | **Without (n=1430)** | **P Value** |
| Age, yrs | 83 (79, 86) | 83 (80, 87) | 83 (79, 86) | 0.152 |
| Female | 1217 (57.2) | 391 (55.9) | 826 (57.8) | 0.424 |
| Logistic EuroSCORE, % | 15.8 (9.1, 26.7) | 16.4 (9.5, 27.6) | 15.5 (9, 26.2) | 0.311 |
| Mean aortic gradient, mmHg | 41 (31, 53) | 42.5 (33, 53) | 40.5 (31, 52) | 0.042 |
| LVEF, % | 60 (45, 60) | 60 (45, 60) | 60 (45, 60) | 0.002 |
| Hypertension | 1886 (88.6) | 624 (89.3) | 1262 (88.3) | 0.488 |
| Diabetes Mellitus | 645 (30.3) | 192 (27.5) | 453 (31.7) | 0.047 |
| Glomerular filtration rate | 47.7 (35.6, 60.4) | 47.7 (35.7, 60.6) | 47.7 (35.5, 60.4) | 0.956 |
| Coronary artery disease | 1355 (63.6) | 440 (62.9) | 915 (64) | 0.64 |
| Peripheral Artery Disease | 269 (12.6) | 86 (12.3) | 183 (12.8) | 0.747 |
| Cerebrovascular Disease | 432 (20.3) | 131 (18.7) | 301 (21) | 0.214 |
| Pulmonal Hypertension | 1118 (52.5) | 365 (52.2) | 753 (52.7) | 0.849 |
| Previous Myocardial Infarction | 334 (15.7) | 106 (15.2) | 228 (15.9) | 0.642 |
| Previous CABG | 192 (9) | 66 (9.4) | 126 (8.8) | 0.633 |
| Previous Aortic Valve Surgery | 51 (2.4) | 13 (1.9) | 38 (2.7) | 0.258 |
| Procedural |  |  |  |  |
| Predilatation | 459 (21.6) | 213 (30.5) | 246 (17.2) | < 0.001 |
| Postdilatation | 435 (20.4) | 135 (19.3) | 300 (21) | 0.371 |
| Valve types |  |  |  |  |
| Balloon-expandable | 1477 (69.4) | 532 (76.1) | 945 (66.1) | <0.001 |
| Self-expandable | 613 (28.8) | 154 (22) | 459 (32.1) | <0.001 |
| Mechanically-expandable | 39 (1.8) | 13 (1.9) | 26 (1.8) | 0.946 |
| Values are median (interquartile range) or counts (percentage) | | | | |
| VD-AR= Videodensitometric assessment of aortic regurgitation, mmHg = millimeter mercury | | | | |
| EuroSCORE = European System for Cardiac Operative Risk Evaluation, LVEF = left ventricular ejection fraction | | | | |
| CABG = coronary artery bypass graft |  |  |  |  |

**Supplemental Figures**


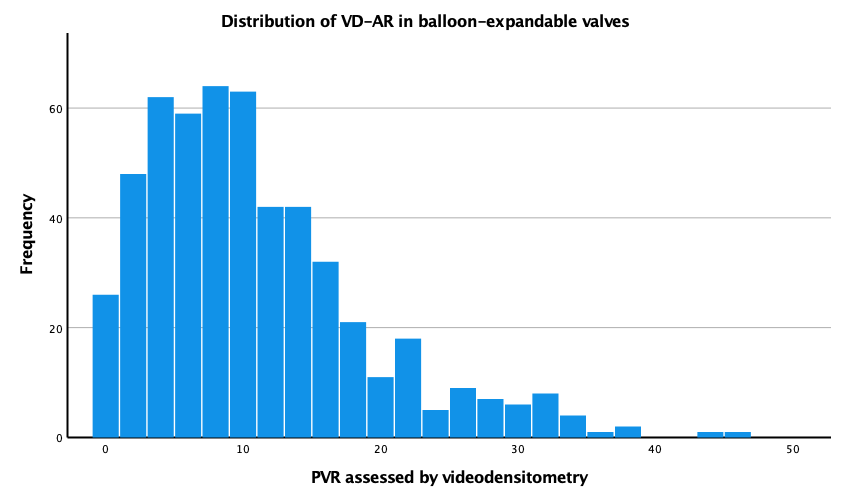

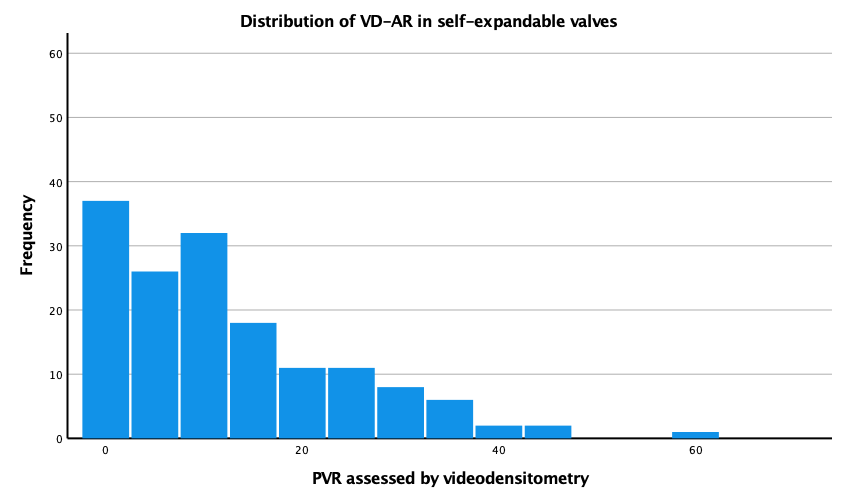

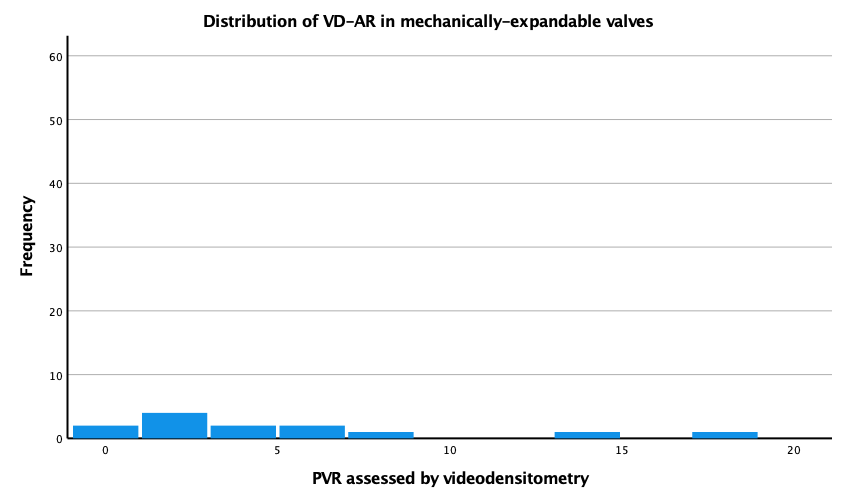


**A**

**C**

**B**

**Supplemental Figure 1.** Distribution of paravalvular regurgitation assessed by videodensitometry (VD-AR) for balloon-expandable valves (**A**), self-expandable valves (**B**) and mechanically expandable valves (**C**). The distribution did not differ significantly between balloon-expandable and self-expandable valves (P = 0.58).
